# Tissue microenvironment induces tissue specificity of ILC2

**DOI:** 10.1038/s41420-024-02096-y

**Published:** 2024-07-16

**Authors:** Minjing Qin, Yuanyuan Fang, Qitong Zheng, Mengyun Peng, Lu Wang, Xia’nan Sang, Gang Cao

**Affiliations:** School of Pharmacy, Zhejiang Chinese Medical University, Hangzhou, China

**Keywords:** Cell biology, Immunology

## Abstract

Type 2 innate lymphoid cells were found to be members of the innate immune cell family, which is involved in innate and adaptive immunity to resist the invasion of foreign antigens and induce allergic reactions caused by allergens. The advancement of ILC2 research has pointed out that ILC2s have a high degree of diversity, challenging the notion of their homogeneity as a cellular population. An increasing number of studies indicate that ILC2 is a cell population with tissue specificity which can be induced by the tissue microenvironment. In addition, crosstalk between tissues can change ILC2 functions of migration and activation. Here, we emphasize that ILC2 undergoes adaptive changes under the regulation of the tissue microenvironment and distant tissues, thereby coordinating the organization’s operation. In addition, ILC2 alterations induced by the tissue microenvironment are not limited to the ILC2 cell population, and ILC2 can also transdifferentiate into another class of ILC cell population (ILC1 or ILC3). In this review, we summarized the tissue-specific effects of ILC2 by tissue microenvironment and focused on the function of ILC2 in inter-tissue crosstalk. Lastly, we discussed the transdifferentiations of ILC2 caused by the abnormal change in tissue environment.

## Facts

1. ILC2s play an essential role in both innate and adaptive immunity.
2. ILC2 derived from different tissues shows significant differences in phenotype due to exposure to different tissue microenvironments.
3. The tissue crosstalk can be caused by ILC2 migration.
4. The distal or proximal tissue microenvironment can induce the transformation, activation, and inhibition of tissue-resident ILC2.

## Open Questions

1. Can drugs treat tissue injury caused by ILC2s by targeting the tissue-specific phenotype of ILC2?
2. Whether ILC2 in different tissues is functionally significantly different from each other?

## Introduction

ILC2s, as members of the ILC family, play an important role in innate and adaptive immunity. Previous studies showed that ILC2s are deeply involved in maintaining tissue homeostasis, activating immunity, and regulating metabolism [1–3]. They rapidly respond to replay early innate immune responses on pathogen infection and tissue damage by directly delivering signals to hematopoietic and non-hematopoietic cells [4, 5]. Although lacking adaptive antigen receptors, ILC2s participate in the regulation of adaptive immune response by secreting immunomodulatory cytokines to direct the immune reaction of helper cells and expressing major histocompatibility complex (MHC) class II to deliver antigen information to adaptive immune cells [6, 7].

Previously, ILC2s were considered homogeneous cells marked with transcription factors: GATA3 and RORα [8]. However, research on both human and mouse ILC2s has revealed that ILC2s are a heterogeneous population of cells with different gene expressions. The transcriptional profile, phenotype, and function of ILC2s can change with the microenvironment in which they reside. These phenomena prompt that the physiological effects of ILC2s are also different [9, 10]. Due to differences in cell composition, cytokine levels, chemokine levels and inflammatory factor levels in the living microenvironment, resident ILC2 possesses heterogeneity between organizations. This character has been called tissue specificity [11]. At the same time, ILC2s can change their transcriptional profile, phenotype and even function to adapt to the tissue environment for exerting effective immunity. Tissue-resident ILC2s exhibit differences in immune responses, including the degree of response to cytokines, conditions required for the secretion of type 2 cytokines, and the function of tissue repair, and so on [12]. In addition to being influenced by the tissue microenvironment, ILC2 can also accept signaling factors that undergo regulation from distal or proximal tissues [13]. And ILC2s also interact with distal tissues to participate in the tissue immune responses [13, 14]. ILC2 is a highly plastic cell population. Tissue ILC2s not only have the potential to transform into other ILC2 subtypes but also transdifferentiate from one subset into another [15]. Studies have revealed that the tissue environment contributed to ILC2 transdifferentiation. However, the abnormality of ILC2 in transdifferentiation can amplify tissue inflammation and induce unprotective type 1 or 3 immune responses [16].

In this review, we elucidate that the tissue microenvironment mainly induces the tissue-specific formation of ILC2 by producing cytokines and influencing the differentiation of congenital lymphoid progenitor cells. Then, we listed recent research about the tissue specificity of ILC2 derived from various tissues, which revealed that ILC2s in different tissues had differences in gene profile, transcriptional profile, and phenotype. Considering the regulatory effect of distal tissues on other tissue ILC2s, we also summarized the latest experimental results, which have found the crosstalk between different tissues can be achieved by influencing the development of ILC2. Then we discussed the transdifferentiation of ILC2 induced by tissue environment and the effect of abnormal ILC2 transdifferentiation.

## Characteristic of group 2 ILCs

**Fig. 1:**
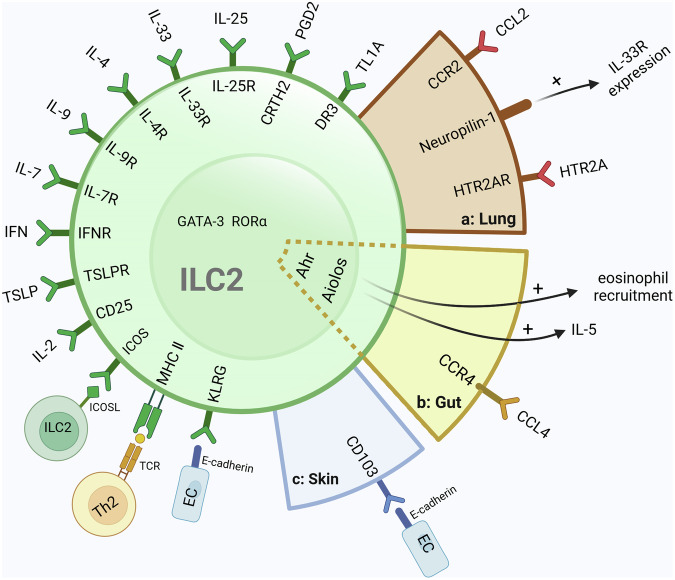
Intrinsic features and tissue-specific features of ILC2. Intrinsic features and tissue-specific features of ILC2 ILC2 commonly expresses the transcription factors of GATA3 and RORα, alarmin receptors (IL-33, IL-25, and TSLP), cytokine receptors (IL-2, IL-4, IL-9, IL-7), lipid mediator receptors (CRTH2, DR3), receptors involved in cellular interactions (ICOS, KLRG), IFN receptors (IFNR), CD25, and MHC II. **a** CCR2, Neuropilin-1, and HTR2AR are tissue-specific markers of lung ILC2. **b** Ahr, Aiolos, and CCR4 are tissue-specific markers of gut ILC2. DR3, member of the TNF-receptor superfamily (**c**) CD103 is a tissue-specific receptor for skin ILC2.; EC epithelial-cell, TCR T cell antigen receptor.

ILCs derive from common lymphoid progenitor (CLP) and can be simply divided into three groups: group 1 ILCs, group 2 ILCs, and group 3 ILCs, based on the differential requirement of transcription factors, the production of signature cytokines, the special phenotypic markers and distinctive effector function [4, 17]. ILC2 is a single subset in group 2 ILCs, and its development depends on the activation of transcription factors: GATA-3, Bcl11b, RORα, TCF- 1, and Gfi1 [18, 19]. ILC2s can be activated by myeloid-cell- or epithelial-cell-derived cytokines (TSLP, E-cadherin, TL1A), alarmins (IL-25, IL-33) and inflammatory mediator (IL-2, IL-4, IL-7, IL-9). Then ILC2s generate a host of cytokines, including IL-5, IL-6, IL-9, and IL-13, to protect against pathogens and induce eosinophilic inflammation by associating type 2 immune response [6]. (Fig. 1) According to previous studies, ILC2s also exert essential effects on repairing tissue damage by amphiregulin production, improving proliferation and differentiation of epithelial cells and further restoring barrier integrity and airway remodeling [20]. Although ILC2s have been proven to have a positive effect on sustaining tissue homeostasis, ILC2 plays an important role in promoting the development of allergic disease and inflammation in the lungs, kidneys, et al., which ultimately result in tissue damage and functional dysregulation. In particular, the lung-resident ILC2s have been revealed to be deeply implicated in promoting pathologic inflammation in allergic asthma through activating Th2 cells and releasing IL-5 and IL-13 to recruit eosinophils and increase mucus production [21]. Nowadays, there is various research on inhibiting activation and immune responses of ILC2s for ameliorating allergic asthma [22–24]. However, ILC2s may not refer to a single-cell population. Huang et al. divided ILC2s into ST2^-^17RB^+^ inflammation ILC2s (iILC2s) and ST2^+^17RB^-^ nature ILC2s (nILC2s) and described the difference between the two groups. nILC2s maintain a stable state in tissue and quickly respond to IL-33. On the contrary, iILC2s are undetectable in a steady state and mainly respond to IL-25 [25]. What’s more, Cai et al. have proved that ST2^+^ ILC2s exist ST2^+^IL17^+^ ILC2s subset, which produced adequate IL-17 to promote lung inflammation, and IL17^-/-^ ILC2s have little effect on this disorder [26]. As mentioned above, ILC2s not only induce allergic diseases but also maintain tissue homeostasis and repair tissue. Excessive inhibition of ILC2 may result in tissue immune disorders, thereby affecting the dysfunction of other tissues and even the whole body. Therefore, the question of identifying the main pathogenic subsets in ILC2 and targeting those cells for treating autoimmune diseases should also be of concern.

## ILC2s involve both innate immunity and adaptive immunity

ILC2, a type of cell involved in the initial immune response, is both closely related to innate and adaptive immunity. ILC2 mainly plays a role in recruiting inflammatory cells, secreting type 2 inflammatory factors, and repairing tissues in innate immunity, while in adaptive immunity, ILC2 mainly promotes T cell activation and proliferation.

### The effects of ILC2s on innate immunity

**Fig. 2:**
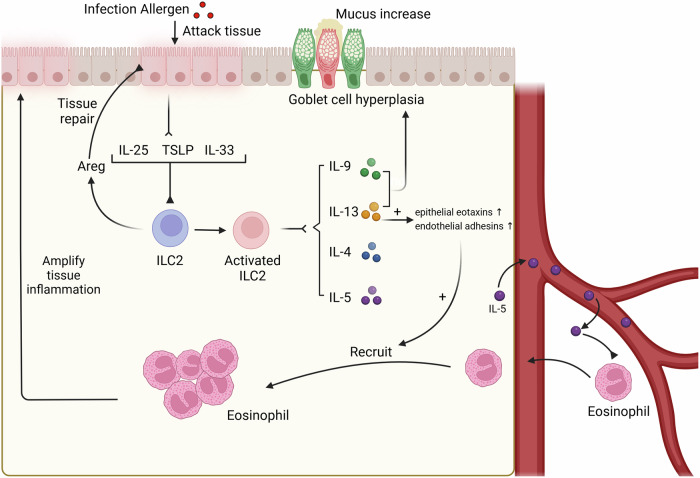
The effects of ILC2s on innate immunity. The effects of ILC2s on innate immunity When infection antigens attack the tissue mucosal barrier, epithelial cells secrete IL-25, TSLP, and IL-33 to activate ILC2 and promote ILC2 to produce Areg. Amphiregulin (Areg) has the function of repairing tissue damage. Activated ILC2 secretes IL-9, IL-13, IL-4 and IL-5. IL-13 induces epithelial eotaxins and endothelial adhesins to promote the recruitment of eosinophil. IL-5 induces eosinophil recruitment in tissues, which could amplify tissue inflammation. IL-9 promotes goblet cell proliferation and increased mucus.

As we know, ILC2s are distributed within the barrier and play an important role in innate immunity, being one of the initial responders of pathogen invasion (Fig. 2). With the stimulation of alarm factor of IL-33, IL-25 and TSLP induced by allergens or helminth, ILC2 can secrete type 2 cytokines, IL-4, IL-13 and IL-5, to participate in innate immune responses [27]. IL-13 can induce epithelial eotaxins and endothelial adhesins necessary for eosinophil trafficking and promote goblet cell proliferation and mucus secretion to facilitate worm elimination [28, 29]. Due to the function of IL-5 in promoting activation, recruitment, and survival of eosinophils, IL-5 secreted by ILC2 supports the involvement of eosinophils in the early phase of allergic inflammation [30, 31]. IL-9 has been proposed to induce mucous production, goblet cell hyperplasia and airway remodeling and plays an important role in the development of allergic lung disease via promoting the production of IL-5 and IL-13 in ILCs. ILC2 not only promotes the activation and aggregation of immune cells but also has the function of tissue repair and maintaining tissue homeostasis. In addition, ILC2 has been confirmed to have the potential to release IL-9, further fostering the resolution of inflammation and restoring immune homeostasis in chronic inflammatory diseases [32, 33]. Under the stimulation of epithelial alarm factor IL-33, ILC2 can produce amphiregulin (AREG) with tissue repair and immune tolerance functions [34]. According to a host of studies, the epidermal growth factor (EGF)^-^like molecule AREG plays a critical role in type 2-mediated resistance and tolerance, which could reduce autoimmune attacks caused by worm or non-worm infections, alleviate local tissue inflammation, promote tissue repair, and maintain the integrity of tissue [35].

### The effects of ILC2s on adaptive immunity

**Fig. 3:**
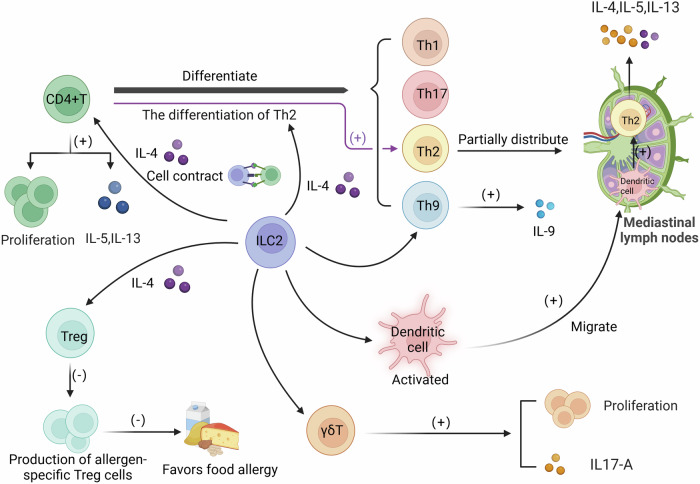
The effects of ILC2s on adaptive immunity. The effects of ILC2s on adaptive immunity ILC2 promotes immune responses of CD4+ T cells, Treg cells and γδ T cells. ILC2 promotes CD4+ T cell proliferation and secretion of IL-5 and IL-13 through secretion of IL-4 and cell contract. CD4+ T cells can differentiate into Th1, Th2, Th17 and Th9 cells. ILC2-derived IL-4 promotes the TH2 differentiation. ILC2 promotes the migration of activated DC cells to mediastinal lymph nodes to activate Th2 cells to secrete IL-4, IL-5, and IL-13. Th9 can be activated by ILC2 to produce IL-9. Production of allergen-specific Treg cells can be inhibited by IL-4, which could suppress favors food allergy. γδT cells can be activated by ILC2 to proliferate and produce IL-9.

ILC2 not only plays an essential role in innate immunity but also has the function of promoting adaptive immunity, mainly reflected in promoting T cell proliferation and activation to enhance adaptive immune effects. (Fig. 3) ILC2s can promote T cell proliferation and type 2 cytokine production (including IL-5 and IL-13) of CD4^+^ T cells via cellar contact and secretion of IL-4 [36]. Moreover, Oliphant et al. found in depleted-ILC2 mice that the ability of CD4^+^T cells to produce IL-13 and IL-5 and expulsion of *N. brasiliensis* was impaired, and revealed that ILC2 promotes CD4^+^T cell proliferation and activation through antigen induction caused by expression of MHC II and present antigen to the T cell. The promoting effect of ILC2 on T cell immune response has also been confirmed in vitro coculture of human-derived ILC2 and CD4^+^ T cells [37]. After the initiation of the adaptive immune response, native CD4^+^ T cells will differentiate into helper T(Th) cells, including Th1, Th2, Th9, Th17, etc, to resist antigens. And ILC2 is closely related to the production and activation of Th2 cells [38]. Th2 cells are the core of type 2 immunity, and their activation is initiated by the recognition of antigens on antigen-presenting cells [39]. ILC2s serve as an innate counterpart of adaptive effector Th2 cells, which can be directly activated by alarmins to produce type 2 cytokines. Although Th2 cells can be activated independently of ILC2, the activation of ILC2s enhances Th2 cell production and immune response [40]. Recent studies have shown that ILC2s promote Th2 cells to participate in type 2 immunity by promoting Th2 cell differentiation. Pelly et al. found that Th2 cell differentiation in mice infected with H. polygyrus depended on ILC2-derived IL-4 [41]. In addition, ILC2 can promote the migration of activated DC to the mediastinal lymph nodes to induce native CD4^+^ T cells to differentiate into Th2 cells [42]. There are also studies indicating that ILC2 can amplify the activation of Th9 cells, but the specific mechanism has not yet been revealed [43]. In addition to participating in the immune response of CD4^+^ T cells, ILC2 can also act on Treg cells and γδT cells to exacerbate allergic inflammation. IL-4 production by ILC2 promotes the food allergic response by reducing allergen-specific Treg cells and activating mast cells [44, 45]. Moreover, ILC2s play a key role in the activation of tissue-specific immune responses in the lung and adipose. OX40L expression by ILC2s is required for IL-33 to drive tissue-restricted Th2 and Treg cell expansion [46]. ILC2s activated by NUM contribute to IL-17-producing γδT cells proliferation and secretion of IL-17A [47].

## The tissue-specific features of ILC2

At first, people thought that ILC2 was a homogeneous cell group that expressed specific transcription factors and cytokines. However, a large number of experiments have shown that ILCs, especially ILC2s, exhibit heterogeneity in phenotype, and function [48]. In recent years, studies have found a unique subset of ILC2 cells that can secrete IL-10, an anti-inflammation factor, in both mice and humans, which possess the effect of inhibiting immune cell proliferation [49, 50]. Transcription data indicated that the gene expression of IL-10^+^ ILC2 is different from that of IL-10^-^ ILC2. Its expression of *Retnla* is upregulated, which is Th2 response negative regulatory factor. Meanwhile, the expression of *Tnf*, *Lta*, *Il2*, and *Ccl1*, pro-inflammatory factors, is downregulated, indicating that it has a different anti-inflammatory effect from other ILC2 [49]. Interestingly, intestinal ILC-derived IL-10 is mainly derived from IL-10^+^ILC2s rather than ILCregs, which have been shown to be similar to Treg cells and participate in intestinal IL-10 secretion [51]. Other studies also revealed that ILC2 subsets that express c-kit markers have distinct transcription and function. c-kit^lo^ ILC2 subset displayed greater potential to produce type 2 cytokines, while c-kit^hi^ ILC2 exhibited an ILC3-like signature, which coexpressed the marker and cham chemokine receptor CCR6 of ILC3 and was able to produce a significant IL-17A under ILC3-promoting condition [15, 52]. Moreover, Huang et al. discovered a new ILC2 subgroup, inflammation ILC2, in the lungs of mice treated with IL-33. Their research suggests that iILC2 is different from the ILC2 that naturally resides in the lung, which has the ability to migrate and is involved in the defense of early worm invasion in the lung [53, 54]. The heterogeneity of ILC2 is not only reflected between ILC2 subpopulations but also between different tissues. Numerous studies have shown that ILC2 derived from different tissues exhibit significant differences in gene profile, transcriptional profile, and phenotype due to the presence of different microenvironments and stimulation of biological signals [55]. These differences are even greater than those between ILC2, ILC1, and ILC3 [11]. The following will provide a detailed introduction to the tissue specificity of ILC2 in different tissues.

### The tissue-specific features of lung ILC2

The lung-resident ILC2s exhibit tissue-specific features in phenotypic receptors and expression of regulators. (Fig. 1a) As mentioned above, ILC2 can be directly activated by alarm factors, IL-25 and IL-33, to participate in the initial type 2 immune response. However, lung ILC2s show differences in the degree of response to cytokines due to the expression of cytokine receptors. Steady-state lung ILC2s highly express ST2 (IL-33 receptor) and produce type 2 cytokines following the stimulation of IL-33 [56], but these ILC2s hardly responded to IL-25 alone because they expressed IL17RB (IL-25 receptor) at a low level [57]. However, cysteinyl leukotrienes secreted by tuft cells can synergize with IL-25 to activate ILC2s and further highly polarize type 2 immune responses [58]. Compared to the transcriptional profile with tonsil and blood ILC2, lung ILC2 derived from humans has a high presence (35%) of CRTH2^-^ ILC2, which could not be found in tonsil or blood ILC2. Moreover, lung ILC2 are more activated and express higher receptors for the ILC2-activating alarmins IL-33 and IL-25 than tonsil and blood ILC2. Interestingly, the expression of IL-1RL1 and IL-17RB was increased in blood ILC2, and the expression of CRTH2 was downregulated after exposure to ILC2-activating alarms, including IL-2, IL-25, IL-33, and TSLP, which demonstrated that the phenotypic changes related to tissue microenvironment [11]. Zao et al. reveal that CCR2 serves as a tissue-specific marker in lung ILC2 and may play an important role in ILC2 re-localization from Bone Marrow (BM) to the lung [54]. Neuropilin-1 is also a tissue-specific marker of lung ILC2, which can enhance ILC2 pro-inflammation function and type 2 immunity by upregulating ST2 expression. Another research points out that Nrp1 in lung ILC2 is closely related to pulmonary fibrosis [55, 59]. Notably, because the receptors for ILC2 in tissues are partly tissue-specific, these receptors may become targets for treating excessive type 2 immunity in the tissues. Wang et al. found HTR2A was highly expressed in ILC2s from mouse lungs and human PBMC. Then, they use antagonists targeting HTR2A receptors in lung ILC2s to suppress type 2 lung immune response induced by ILC2 activation [60]. In addition to the tissue-specific features, lung ILC2 is heterogeneous and the single population of lung ILC2 may exert different functions in immunity and tissue repair. In a 2019 experiment, RORα- YFP mice, whose cells express *Rorα* during their development and can be irreversibly labeled by Yellow Fluorescent Protein (YFP), were used to analyze the heterogeneity of lung ILC2s. RORα-YFP ILC2 from adult mouse lung could be divided into two subsets according to the difference in expression level of ILC2-associated genes (*Il1rl1, Tnfrsf18, Areg, Arg1, Cd7, Runx3, Cd2, Tcf7, Il18r1*). Moreover, distinct effector ILC2 subsets (KLRG^+^ICOS^-^ and KLRG^-^ICOS^+^ ILC2 subsets) in neonatal mouse lungs were discovered, which mainly produced pro-inflammation cytokines (IL-5 and IL-13) or amphiregulin promoting tissue repair [61].

### The tissue-specific feature of gut ILC2

Aryl hydrocarbon receptor (Ahr) is a ligand-dependent environment sensor that was thought to be part of the tissue adaptation mechanism in cells promoting the host to adapt to external environmental changes. (Fig. 1b) Compared to ILC2s in the lung, fat, mesenteric lymph nodes, skin, or blood, gut ILC2s in mice highly express Ahr, and the expression of Ahr in gut ILC2 was supported by the gut environment. Interestingly, Ahr could suppress ILC2 function, inhibiting the expression of IL-33 receptor and effector molecules IL-5, IL-13 and amphiregulin. This finding indicated that the gut ILC2s process tissue-specific features and the function of gut ILC2s may differ from other ILC2s distributed in other tissues or blood [62]. Jinxin Qiu et al. not only confirmed Ahr to be an intestine ILC2s feature but also found *Ikzf3* encoding the transcription factor Aiolos as the tissue-specific feature by investigating the differential expression of the gene in mice and humans. (Fig. 1b) Aiolos can regulate the function of immune cells, including ILC2s, which can sustain IL-5 expression in ILC2s and promote eosinophil recruitment by inhibiting PD-1. Notably, this study also found that Ahr plays an important role in the long-term maintenance and IL-5 production of ST2^+^ ILC2s through sustaining Aiolos expression and inhibiting PD-1. The regulation of Aiolos expression by Ahr in ILC2s implies that the mechanism of maintaining functions of ILC2s in the intestine is different from other tissue ILC2s [12].

### The tissue-specific feature of skin ILC2

The skin is the outermost barrier between the environment and internal organs that contain cells of the immune system deployed as sentinels that serve as a first line of defense against microbial attacks. As one of the components of mucosal immunity, the skin also contains ILC2 cells. A previous study proved that a population of CD45^+^CD11b^-^CD90^hi^CD3^-^CD2^-^ ILC2s exerted in wild-type mice dermis, which uniquely expressed CD103 and lacked some of the characteristic markers of ILC2s, such as CD25, Sca-1, CD117 and ST2, being different with other mucosal ILC2s [63].(Fig. 1c) And the expression proportion of skin ILC2 cytokine receptor also has tissue specificity. Skin ILC2 all expresses IL-18Rα, which differs from ILC2 groups from the lung and bone marrow, where only 5% -10% of ILC2 express IL-18 receptors. IL-18 was an essential cytokine to proliferate skin IL-18Rα^+^ST2^-^ ILC2 and produce IL-5 and IL-13 in a model of atopic dermatitis. However, IL-18 cannot effectively activate lung *Il5*^*+*^ ILC2s to produce IL-13 [61]. Single-cell sequencing was used to detect ILC2 sorted from the skin, revealing that the transcription profile of skin ILC2 was significantly different from ILC2 in the bone marrow and other peripheral tissues. Although skin ILC2s broadly express *Gata3*, *Il7r*, and *Crlf2*, the expression of *Icos*, *Ccr6*, and *Itgae* is highly enriched in skin ILC2s and expression of *Il1rl1* in skin ILC2s is less than lung and adipose ILC2s [64]. Moreover, the phenotype and function of skin ILC2s can be induced to change when the microenvironment is changed by diseases. In psoriasis induced by IL-23 or imiquimod, the resting ILC2 population in the skin can specifically transform into an ILC3-like subset and participate in the immune response of psoriasis to promote skin inflammation [15].

## The relation between tissue microenvironment and tissue-specific in ILC2s

ILC2s possess the ability to change their function to adapt to various tissue microenvironments, which contributes to tissue ILC2s having tissue-specific characteristics. Many researchers have reported that tissue microenvironments can induce ILC2 to acquire tissue-specific features through cytokines and the differentiation of ILC progenitor cells (ILCP).

### Tissue microenvironment promotes the differentiation of ILCP into tissue-specifical ILC2

ILC progenitors can seed tissues during the prenatal period [65]. And ILC2 can be found in various tissues during late gestation. However, a majority of peripheral ILC2s were distributed in tissues during the post-natal window and quickly expanded and differentiated to consist of tissue-resident ILC2 pools. This period was accompanied by the acquisition of tissue-specific transcriptomes, which suggests that the tissue specificity of ILC2 can be shaped by the differentiation of early immature ILC2 induced by the tissue microenvironment in which ILC2s were located [66]. In addition to deriving from the naive ILC2s that were generated in the period from birth through weaning, tissue ILC2s can be produced by the differentiation of local ILCPs and mature in the tissue. Naive Il18r^+^ST2^−^ ILCs, the local ILCP with differentiation ability, were found in mice and humans’ lungs, contributing to the expansion of lung ILC2s. What’s more, when lungs were infected by *N. brasiliensis*, BM Il18r^+^ ST2^−^ILCs were recruited into the lungs and can generate the entire spectrum of lung ILC2s based on their single-cell transcriptomes. These results imply that ILCP, located in various tissues, retains the potential to differentiate into tissue ILC2s, and this differentiation process may be guided by the tissue microenvironment [67]. In addition, studies have found a type of ILC precursor cell, CD62L^+^ ILCP, which mainly differentiates into ILC2. However, in psoriasis patients, CD62^+^ ILCP is reduced, while the number of CD62^-^ is upregulated, and these cells tend to differentiate into ILC3s, which enrich in psoriasis. This research indicated that the proliferation of precursor cells of ILC2 is influenced by the disease environment [68].

### Cytokines in tissue microenvironment support phenotypic changes in mature ILC2

With the development of research on tissue ILC2, it has been gradually discovered that the secreted products of tissue can promote the expression of tissue-specific phenotypes in ILC2. As we know, resting IL-5^+^ ILC2s required the stimulation of epidermal cytokines, IL-33, IL-25, and TSLP, to produce type 2 cytokines [69]. However, in the mice triple-deficient in TLSP receptor, IL-33 receptor, and IL-25, it was found that ILC2 can produce IL-5 in the skin, indicating that skin microenvironment exists factors to maintain the immune function of ILC2 in the skin. According to the transcriptomic signatures imprinted by each tissue, the expression of *Il18αr* in skin ILC2 is significantly higher than ILC2s in other tissues. And it has been proved that the presence of IL-18 can enhance the production of IL-13 in skin ILC2s and maintain the steady-state activation of skin ILC2s. The article points out that factors derived from different tissues contribute to the activation of the ILC2 subgroup in the steady state. Later, this study also conducted transcriptional profiling analysis on various tissues, such as the lung, fat, and gut, and found that ILC2 from different tissues was tissue-specific and independent of epithelial cytokines [70]. The phenotype of tissue ILC2 can change with changes in the culture environment. Qiu et al. has found the phenotypes of ILC2 from gut, pancreas and lung changed while respectively cultured with tissue cells that were different from the source of tissue ILC2. Then they indicated that the tissue microenvironment actively shaped ILC2 characters [12]. In addition, studies have shown that Nrp1 is a specific marker of lung ILC2, playing an important role in ILC2 function, and the expression of Nrp1 is induced and maintained by the lung microenvironment. This experiment co-culture ILC2 isolated from the lungs and intestines with monocytes from lung and intestinal tissues to explore the adaptation of tissue ILC2 to environmental cues. The Nrp1 expression of intestinal ILC2 co-cultured with lung cells was upregulated, while the Nrp1 expression of lung ILC2 co-cultured with intestinal cells was inhibited. These findings demonstrated that the expression of Nrp1 required lung environment signals to induce and maintain. Compared with other tissues, lung has the highest expression level of TGF-b1, which is the ligand of TGFβ1. And TGFβ1 has been confirmed to have a direct relationship with the maintenance of Nrp1 expression [55].

## ILC2 functions be regulated by distal or proximal tissue microenvironment

Interestingly, in addition to being influenced by tissue microenvironments, ILC2 also can interact with distal or proximal organs. This tissue crosstalk not only can be caused by ILC2 migration but also induces the transformation, activation, and inhibition of tissue-resident ILC2.

### Alterations in the tissue microenvironment induce ILC2s to acquire migratory capacity

**Fig. 4:**
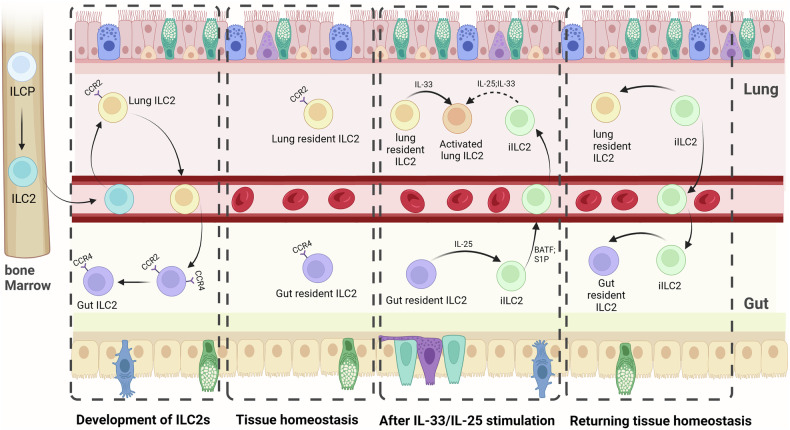
Migration of ILC2s between lung and intestine. Development of ILC2s: CCR2^+^ ILC2 in the lung is initially derived from bone marrow ILC2 differentiated from ILCP; during development, lung ILC2 migrates to the intestine and the phenotype shifts from CCR2^+^ CCR4^−^ to CCR2 ^+^ CCR4^+^ and then to CCR2^-^CCR4^+^. Tissue homeostasis: in a stable tissue microenvironment, lung-intrinsic ILC2 stably expresses CCR2 but not CCR4, and intestinal-intrinsic ILC2 expresses CCR4 but not CCR2. After IL-33/IL-25 stimulation: when intestinal ILC2s is stimulated by IL-25 it differentiates into iILC2; iILC2 relies on BATF and S1P for migration into the lung; Lung intrinsic ILC2 and iILC2 are activated by stimulation with IL-33 or IL-25. Returning tissue homeostasis: after the immune response subsides, some of the iILC2s transform into lung-intrinsic ILC2 and other parts of the ILC2s return to the small intestine through the circular system.

Initially, ILCs were identified in both lymphoid and non-lymphoid organs as tissue-resident cells, and the tissue residency of ILCs, including ILC2s, was even maintained under systemic inflammation created by the deficiency of FOXP3 in mice [71]. However, recent studies have shown that the resident-ILC2 in lung and gut can migrate between lung and gut, which is accompanied by the subset transformation of ILC2. Huang et al. identified another cell subtype of ILC2 distinct from nILC2: iILC2, which is thought to be a transient progenitor of inflammatory and infective ILCs that replenish nILC2 and contribute to immunity against worms and fungi [53]. And a subsequent study by Huang et al. found that nILC2 in the gut can proliferate and transform to migratory iILC2 after being activated by IL-25. The iILC2s migrated similarly to T lymphocytes. They depended on S1P entering lymphatic vessels and then entering the circulatory system, which mostly raised in the lung to exert effects of tissue repair and anti-helminth infection [13]. (Fig. 4) In addition to the migration of ILC2 from gut to lung, a paper in 2022 indicated that ILC2 can migrate and develop from lung to gut. Zhao et al. found that the ILC2 in the lung and gut respectively express CCR2 or CCR4, and took the expression level of *GATA3* and *Klrg1* as maturity index to detect the maturity degree of ILC2 from the gut to the lung. Then, they found that ILC2, migrating from the lungs to the intestine, matures gradually from lung to intestine. Notably, further research on ILC2 development revealed that lung CCR2^+^ ILC2 produced gut CCR4^+^ ILC2. And it was discovered that the CCR2^+^CCR4^+^ ILC2 existed in the gut, suggesting that these ILC2s may have undergone a transition from CCR2^+^ ILC2s to CCR4^+^ ILC2s. (Fig. 4) The above findings indicated that lung ILC2 gradually migrates and develops into the intestine, and subtypes change during migration to adapt to the intestinal environment [54]. The microenvironment change can not only induce migration of ILC2 to other tissues but also promote migration of ILC2 from the adventitial niches to tissue parenchymal sites. Cautivo et al. found activation of type 2 immunity induced by IL-33 or infection with *Nippostrongylus brasiliensis* helminths could induce lung IL-5^+^ ILC2, distributing in adventitial niches around larger vessels and airways, migrate to de novo parenchymal sites. This phenomenon is also observed in the liver and perigonadal adipose tissue, innate and adaptive type 2 responses leading to the expansion and appearance of IL-5^+^ ILC2 within parenchymal areas in proximity to hepatocytes. And the restriction of ILC2 migration to tissue parenchyma can be induced by the type I immune factor IFN- γ, which causes a reduction in type 2 inflammation and worsening of worm infection. The authors stated that the regulation of ILC2 migration within organizations was a critical determinant of beneficial and pathologic organ inflammation and repair [72]. Moreover, the migration of ILC2 also contributes to systemic inflammation. It has been found that cutaneous local innate responses can be amplified to systemic type 2 responses by activated ILC2s migrating from the skin into the draining lymph node [73].

### The functions of ILC2 are influenced by mucosal neuro-lung crosstalk

Neuronal signals have emerged as pivotal regulators of ILC2s that regulate tissue homeostasis and allergic inflammation. Neuromedin U (NmU) is a group of neuropeptides secreted by neurons located near immune cells, which has been proven to activate immune cells directly and play a critical role in neurogenic inflammation [74]. Previous studies revealed that the gene *NMUR1* was selectively enriched in mouse ILC2s and human ILC2s also express NMUR1, which indicated that ILC2s could accept the stimulation of NMU. And the expression of pro-inflammation and tissue-protective type 2 cytokines genes, *IL5, IL13*, Areg and colony-stimulating factor 2 (*Csf2*), increased after lung-derived ILC2 was activated with NMU. Notably, the ILC2-autonomous ablation of *Nmur1* induced downregulation of type 2 response and reduced the immune resistance to worm infections. In septic mice, NUM was also critical for promoting IL-17A- producing γδ T cell expansion and expression of IL-17A by activating lung ILC2. These researches indicated that the NMU-NMUR1 axis plays an important role in the production of ILC2-derived cytokines, and paracrine neurons can direct the sense of exogenous antigen and alarmins and produce NMU to enhance innate type 2 immunity for providing immediate mucosal protection [47, 75]. What’s more, NMU can amplify the stimulation of alarmin (IL-25) to ILC2s. According to previous research, lung ILC2s can be activated to proliferate and highly express IL-13, the effect factor of ILC2, after being treated with IL-25 and NMU [76]. Interestingly, the nerve signals not only enhance ILC2 function but also suppress ILC2 activation. Dopamine, an essential neurotransmitter that relates to immune cell activity and alleviates inflammation [3], can inhibit lung ILC2 responses in allergic airway inflammation by impairing the function of the mitochondrion in ILC2 via the oxidative phosphorylation pathway [77]. Neuropeptide CGRP suppresses IL-33-induced pulmonary inflammation and worm expulsion of *N. brasiliensis* by inhibiting the immune response of ILC2s. Interestingly, CGRP plays a role in slamming on the brake of activation of ILC2s by NMU. CGRP receptor expression increased at a later time point following infection, whereas Nmur1 expression in ILC2s was downregulated. The results show that the nervous system can participate in the regulation of lung immunity by stimulating or suppressing the activation of lung ILC2 through secreted nerve signals [78].

## Tissue microenvironment induces the transdifferentiation of ILC2s

Recent studies have shown that under the influence of diseases, the composition of tissue ILCs changes due to the transdifferentiation of one ILC into another, including the transformation of ILC2 into ILC1 and ILC3, which can further disrupt tissue function. The transdifferentiation of ILC2 is similar to the transformation between ILC2 subgroups, which were accompanied by the change of transcriptional profile, phenotype, and function. Research has shown that lung ILC2s isolated from chronic obstructive pulmonary disease (COPD) could transdifferentiate into ILC1s when stimulated by IL-β and IL-12, with the reduction of GATA3 transcripts and GATA3 protein expression and upregulation of T-bet expression, while IL-4 can restore the functional characteristics of ILC2. These demonstrated that lung ILC2s had the potential to transdifferentiate into ILC1s when exposed to a type 1 inflammatory environment, such as COPD. Meanwhile, this study also observed that CRTH2^-^ ILC2 from peripheral blood and nasal polyps of patients with chronic sinusitis has low expression of *GATA3* mRNA and high expression of *TBX21* mRNA and *RORC* mRNA, and can secrete IFN-γ in response to type 1 stimulation, which indicated that the occurrence of chronic sinusitis promoted ILC2s to acquire the character of producing IFN-γ ILC1. Therefore, imbalance of IL-4, IL-12 and IL-1β may cause disorder in the transdifferentiation of ILC2 and ILC1, resulting in the perpetuation of type 1 or type 2 inflammation [79, 80]. In 2019, Bernink et al. revealed that ILC2s had the potential to transform into ILC3-like cell population capable of producing IL-17 and referred that the downregulation of GATA-3 expression is an essential factor for ILC2s to transdifferentiate into ILC3 like cells, which can be caused by IL-23 and TGF- β in skin microenvironment. Further, this research compared normal skin specimens with biopsies of patients with psoriasis and found a significant increase in the frequency of NKp44^-^ ILC3s, which was associated with a decrease in CRTH2^+^ ILC2s derived from the dermis of psoriatic lesions, and above results demonstrate that the ILC3 increase within skin lesions of patients might result from an increased shift of ILC2 toward ILC2-derived ILC3s [15]. And this conclusion was proved in 2020 by Bielecki et al. by structuring the topic model of ILC scRNA-seq time in psoriasis induced by IL-23 and using their directed-diffusion method to calculate the time trajectory of ILC3 differentiation. Research data suggest that the resting ILC2 population in the skin can specifically transdifferentiate into an ILC3-like subgroup in psoriasis induced by IL-23 or imiquimod and participate in the immune response of psoriasis to promote skin inflammation [64].

## Conclusion

In contrast to previous views, ILC2s are heterogeneous cells that undergo changes in gene profile, transcriptional profile, and phenotype based on changes in the tissue microenvironment. Tissue microenvironments induce ILC2s tissue-specificity to adapt to the tissue environment and involve tissue immune responses by influencing the differentiation of congenital lymphoid progenitor cells and secreting cytokines. At present, ILC2 subgroups with tissue specificity have been found in several organizations, indicating that the tissue specificity of ILC2 is universal. However, there are still insufficient studies to demonstrate whether tissue heterogeneity of ILC2 has an impact on ILC2 function. In addition, crosstalk of tissues can cause transdifferentiation from intrinsic ILC2 to migratory ILC2, as well as activation and inhibition of ILC2 function, indicating that the regulation of ILC2 in an organization is not only related to the microenvironment of the organization but also to signaling molecules in other tissues. ILC2s are highly dynamic cells that respond, migrate, and transdifferent depending on the microenvironment. Thus, ILC2s are easily dysregulated and caused by abnormal microenvironment, resulting in diseases such as inflammation, allergic disease, and disordered tissue repair. Hence, maintaining the stability and balance of ILC2 should be considered one of the critical factors in maintaining tissue homeostasis.
